# Real‐time evaluation can inform global and regional efforts to improve breastfeeding policies and programmes

**DOI:** 10.1111/mcn.12774

**Published:** 2019-02-22

**Authors:** France Bégin, Karin Lapping, David Clark, Irum Taqi, Christiane Rudert, Roger Mathisen, Zoe Stathopoulos

**Affiliations:** ^1^ Programme Division (Early Childhood Nutrition), UNICEF New York NY, USA; ^2^ Alive & Thrive Initiative FHI 360 Washington DC, USA; ^3^ UNICEF East Asia and Pacific Regional Office Bangkok Thailand; ^4^ Alive & Thrive Initiative FHI 360 Hanoi Vietnam

The authors Michaud‐Létourneau et al. have identified three main drivers that large initiatives should adopt to enhance their effectiveness in advocacy for policy change: (a) the use of an explicit advocacy approach, (b) the creation of a strategic group of actors, and (c) the realization of 15 critical tasks, more specifically related to implementation of the International Code of Marketing of Breast‐milk Substitutes (the Code) (Michaud‐Létourneau, Gayard, & Pelletier, 2019a, 2019b) and relevant resolutions of the World Health Assembly. Moreover, implementation research (Theobald et al., 2018) can provide valuable insights to policy design and implementation. The real‐time assessment shared in this supplement describes a state‐of‐the art evaluation method of advocacy for policy change by measuring the extent to which policy objectives were achieved, and by identifying drivers and triggers of policy change. Learning was also generated during the implementation period prior to endline evaluations, facilitating timely course correction to ensure interventions are adapted to context and more likely to have an impact.

While these drivers have been identified to initiate policy change at the country level, they are also relevant at the global level to facilitate policy change in countries and encourage greater donor investment in breastfeeding programmes. The 2016 *Lancet* Series on Breastfeeding stressed the need for stronger international leadership to coordinate and stimulate strategic action across countries and identify where investment is needed to protect, promote, and support breastfeeding (McFadden et al., 2016).

At the global level, the Global Breastfeeding Collective (“the Collective”) is a partnership led by UNICEF and WHO and comprises more than 20 organizations, including implementers and donors from governments, philanthropies, international organizations, and civil society. The Collective works to accelerate progress towards an environment that enables mothers everywhere to breastfeed. The Collective developed a strategic advocacy approach to unify and align partners around shared priorities, messaging, and A Call to Action; the approach includes seven key policy and programme actions that need to be undertaken to achieve the World Health Assembly's 2025 global breastfeeding target. Specifically, the Collective calls on governments to (a) adequately fund programmes that support breastfeeding; (b) adopt and enforce the Code; (c) enact paid family leave and workplace breastfeeding policies; (d) establish national and health facility‐level policies that promote the Ten Steps to Successful Breastfeeding (UNICEF & WHO, 2018); (e) provide more breastfeeding counselling to all mothers; (f) encourage community support for breastfeeding; and (g) track the data to measure progress on breastfeeding programmes.

To stimulate action and track progress on international breastfeeding targets, the Collective developed a number of advocacy tools, such as an annual Global Breastfeeding Scorecard. This scorecard compiles available data from countries across the globe on the status of key indicators related to the Collective's seven policies and programme actions that impact breastfeeding rates (Global Breastfeeding Collective, 2017a). The latest scorecard highlights that while several countries have made significant progress, there is no single country that has met all the policy recommendations. Globally, the percent of countries carrying out the recommended actions is far too low (Global Breastfeeding Collective, 2018).

Building on the World Bank's Investment Framework for Nutrition (Walters, Eberwein, Sullivan, & Shekar, 2017), an investment case for breastfeeding was also developed. The investment case advances the Collective's call to government officials, policy makers, and donors to increase political commitment and financial investment in breastfeeding programmes and supportive policies. The investment case shows that in China, India, Nigeria, Mexico, and Indonesia alone, the lack of support and investment in breastfeeding programmes accounts for more than 236,000 child deaths every year and almost $119 million per year in economic losses (Global Breastfeeding Collective, 2017b).

Both the scorecard and investment case have been used in country‐level advocacy efforts. In Nigeria, for example, a high‐level event was hosted with the support of the First Lady's office. The event showcased Nigeria's progress against the scorecard indicators and the need for greater investment in breastfeeding programmes, generating significant media attention. Partners in Nigeria are working together in advocating for the expansion of maternity leave at the federal level and for the National Food and Drug Administration to approve legislation to better enforce the Code. More recently, UNICEF Nigeria, Alive & Thrive, and partners began working at the state level with the Ministry of Women's Affairs and Ministry of Health to expand maternity leave and Code enforcement.

In China, the high‐level Breastfeeding Promotion Forum in Beijing during the 2018 World Breastfeeding Week called for more investment in breastfeeding. The need for stronger national measures to implement the Code and alignment of paid maternity leave regulations with the recommended exclusive breastfeeding period was identified as priorities. The National Health Commission has further commenced preparatory work on Code legislation. A National Infant and Young Child Feeding Alliance was recently established by a group of actors, including UNICEF and A&T, to facilitate coordinated support in line with the approach and learning from ASEAN member states described in this supplement. There has also been extensive knowledge exchange between China and Vietnam, including recent learning visits focusing on strengthening Code implementation, paid maternity leave and workplace lactation support, and solutions to create more breastfeeding‐friendly health systems.

Adoption of policies that can impact breastfeeding rates is important, but monitoring and enforcing the policies and legislation is just as critical. Recent data indicate that currently, 136 countries have enacted legal measures related to the Code, but only 35 countries have enacted all of its provisions, and very few have functioning monitoring and enforcement mechanisms in place (WHO/UNICEF/IBFAN, 2018). Clearly, more concerted efforts are required to strengthen policies and their implementation.

Building on the success achieved in working through regional platforms to improve infant and young child feeding policies, strategies, and financing in ASEAN countries as highlighted in this supplement, efforts are now underway to adopt a similar approach to strengthen national Code legislation, monitoring, and enforcement in three additional regions: West and Central Africa (WCA), East and Southern Africa (ESA), and South Asia. Building stronger organizational leadership of regional economic platforms with greater capacity to improve policies, legislation, strategies, and financing is the focus. UNICEF, WHO, Alive & Thrive, International Baby Food Action Network (IBFAN), and Helen Keller International are providing technical assistance, including legal support and training on the Code, monitoring and enforcement systems, and supporting harmonization of technical policy and advocacy for maternity protection legislation. Work has begun on the development of Regional Code and Maternity standards through the ASEAN working group, and agreement has been reached with key partners on a coordinated and synergistic approach. Meanwhile, in ESA, the Southern African Development Community (SADC) and the Intergovernmental Authority on Development (IGAD) have acknowledged that Code implementation, monitoring and enforcement, and improvements in maternity protection are priorities for the region and have committed to support the development and promotion of regional standards. In WCA, work began through a joint regional IBFAN/WHO/UNICEF Code training held in Ouagadougou in September 2019, with participation from nine African francophone countries. This has created awareness and demand for support from participating countries. In South Asia, seven out of eight countries have strong Code legislation in place, and regional efforts will thus focus on establishing effective monitoring and enforcement systems through the WHO/UNICEF NetCode initiative. These regional initiatives are very much needed to complement global advocacy efforts.

It is also critical to build alliances with relevant sectors and groups to expand its reach and collective voice. Building strong connections with the organizations and initiatives working on gender‐related policies and programmes have potentials by advocating that supporting a woman's right to breastfeed is a measure of gender equality and promoting a breastfeeding‐friendly society is a collective responsibility. The Scaling‐Up Nutrition Movement is another example of multistakeholder and multisectoral efforts at global, regional, and country level to improve breastfeeding and nutrition outcomes.

Global and regional initiatives can learn from insights provided by the real‐time evaluation conducted in Southeast Asian countries described in this supplement. For example, plans are underway to evaluate the advocacy efforts of the Collective on its desired outcomes. This should help contribute to building the evidence that is needed on both the effective strategies and the resulting effects of advocacy initiatives on policies and programmes.

## CONFLICTS OF INTEREST

The authors declare that they have no conflicts of interest.

## CONTRIBUTIONS

All authors contributed to the manuscript.

## FUNDING

Alive & Thrive is funded by the Bill & Melinda Gates Foundation, the governments of Canada and Ireland and is managed by FHI 360.
